# Immunoexpression of napsin a in renal neoplasms

**DOI:** 10.1186/s13000-015-0242-z

**Published:** 2015-03-14

**Authors:** Bing Zhu, Stephen M Rohan, Xiaoqi Lin

**Affiliations:** Department of Pathology, Northwestern Memorial Hospital, Feinberg School of Medicine, Northwestern University, 251 E. Huron St., Galter Pavilion 7-132 F, Chicago, IL 60611 USA

**Keywords:** Renal neoplasm, Napsin A, Immunohistochemistry

## Abstract

**Background:**

Immunohistochemistry (IHC) for napsin A has been widely used to support a diagnosis of lung adenocarcinoma with high sensitivity. In this study, we evaluated immunoreactivity for napsin A in a broad spectrum of renal neoplasms by using tissue microarrays (TMA).

**Methods:**

Duplicate TMA of 159 surgically excised renal neoplasms of various types were constructed. IHC for napsin A was performed on TMAs with appropriate positive and negative controls.

**Results:**

Napsin A was expressed in Acquired cystic disease associated renal cell carcinoma (RCC) (2/2, 100.0%), chromophobe RCC (5/45, 11.1%), clear cell RCC (10/23, 43.5%), clear cell papillary RCC (9/19, 47.4%), metanephric adenoma (3/3, 100.0%), oncocytoma (13/23, 56.5%), and papillary RCC (31/37, 83.8%). Expression of napsin A was not seen in mucinous tubular and spindle cell carcinoma (0/1, 0.0%), TFE/MITF RCC 0/1, 0.0%), and urothelial carcinoma (0/6, 0.0%).

**Conclusions:**

Napsin A is expressed in both common and rare sub-types of renal neoplasms with variable sensitivity. Based on our results, napsin A is not specific for lung adenocarcinoma. When a metastatic carcinoma of unknown primary is positive for napsin A, the differential diagnosis should include tumors of both renal and lung origin.

**Virtual slides:**

The virtual slide(s) for this article can be found here: http://www.diagnosticpathology.diagnomx.eu/vs/9558727831304717.

## Background

Napsin A is an aspartic proteinase [1], an enzyme of the pepsin family. Napsin A is expressed in type II pneumocytes and alveolar macrophages of the lung, the proximal and convoluted tubules of the kidney, and acini and ducts of the pancreas [1,2]. Immunohistochemistry (IHC) for napsin A has been widely used to support a diagnosis of lung adenocarcinoma with reported high sensitivity (59% - 100%) [3-6], specificity (88 - 94%) [5,6], positive predictive value (78 - 90%) [5,6], and negative predictive value (72 - 96%) [5-7]. The reported sensitivity and specificity of immunohistochemical labeling for napsin A and TTF-1 for supporting the diagnosis of primary lung adenocarcinoma are controversial due to difference in case number (155 vs. 1674 cases), and tumor area (tissue block vs, tissue microarray) [6,7]. It is well established that distinguishing primary lung adenocarcinoma from squamous cell carcinoma, and neuroendocrine carcinomas (including small cell carcinoma) is clinically important [3,4,7].

In addition to the expression of napsin A in lung adenocarcinoma, immunoreactivity for napsin A has also been documented in 5.3 - 48.3% of papillary thyroid carcinomas [3,4], 79.0 - 87.5% of papillary renal cell carcinomas (RCC) [3,4,7], 29.4 - 52% of clear cell RCC [3,4,7], 3.9 - 20.0% of chromophobe RCC [3,4], 5 - 20% of hepatocellular carcinoma [6,7], and 8 - 20% of endometrial adenocarcinoma [3,7]. Other tumor types, such as squamous cell carcinoma (0 – 3% [7]), oncocytoma [3], colonic adenocarcinoma (0 – 2% [7]), pancreatic adenocarcinoma (0 – 4% [7]), gastric adenocarcinoma, mesothelioma, ovarian carcinoma (0 – 6% [7]), urothelial carcinoma, prostate adenocarcinoma, and breast adenocarcinoma (0 – 3% [7]), have been described as being negative or very rarely positive for napsin A [3,4,6,7].

The new international society of urological pathology (ISUP) Vancouver classification of renal neoplasia classifies renal neoplasms into broad categories including renal cell tumors, metanephric tumors, nephroblastic tumors, mesenchymal tumors, mixed mesenchymal and epithelial tumors, neuroendocrine tumors, hematopoietic and lymphoid tumors, germ cell tumors, metastatic tumors and other tumors [8]. As Napsin A has been shown to be expressed in some types of epithelial neoplasms, in this study, we evaluated the immunoreactivity for napsin A in a broad spectrum of epithelial renal neoplasms classified according to the new ISUP classification—including novel, recently described sub-types.

## Methods

### Patients

This study was approved by our Institution Review Board. One hundred and fifty nine renal neoplasms that had undergone resection at our institution between January 1, 2003 and December 31, 2012 were selected based on the availability of H&E slides and sufficient tissue in the corresponding paraffin blocks to perform the studies outlined herein. All H&E slides from each case were reviewed by a fellowship trained genitourinary pathologist (SMR). The tumors were classified based on the international society of urological pathology (ISUP) Vancouver classification of renal neoplasm [8]. These tumors included 45 chromophobe RCC, 37 papillary RCC, 23 clear cell RCC, 23 oncocytoma, 19 clear cell papillary RCC, 2 acquired cystic disease associated RCC, 3 metanephric adenoma, 1 mucinous tubular and spindle cell carcinoma, 1 RCC associated with an Xp11.2 translocations/TFE3 gene fusion (TFE/MITF RCC), and 6 urothelial carcinomas of the renal pelvis (Table 1).

**Table 1:** **Expression of Napsin A in renal neoplasms**

**Neoplasms**	**No.**	**Napsin A No. (%)**
Acquired cystic disease associated RCC	2	2 (100.0)
Chromophobe RCC	45	5 (11.1)
Clear cell RCC	23	10 (43.5)
Clear cell papillary RCC	19	9 (47.4)
Metanephric adenoma	3	3 (100.0)
Mucinous tubular and spindle cell carcinoma	1	0 (0.0)
Oncocytoma	23	13 (56.5)
Papillary RCC	37	31 (83.8)
TFE/MITF RCC*	1	0 (0.0)
Urothelial carcinoma	6	0 (0.0)

*: TFE/MITF RCC: RCC associated with Xp11.2 translocations/TFE3 gene fusions.

### Construction of tissue microarrays

After review of all H&E stained slides from a given case, a single tumor containing paraffin block was chosen for inclusion in a tissue microarray (TMA). For this study, duplicate TMAs were constructed using 2 mm tissue cores.

### Immunohistochemistry

An immunohistochemical (IHC) stain for Napsin A (Catalog CM388CK, Biocare Medical, Concord, CA) was performed on sections from the TMAs on an automated stainer with appropriate positive (lung adenocarcinoma) and negative controls (colon and prostate adenocarcinomas) [9]. Paraffin-embedded blocks were sectioned, deparaffinized, rehydrated, and blocked with methanolic 3% hydrogen peroxide. Antigen retrieval was performed in citrate buffer. After incubation with the primary anti-Napsin A antibody, the detection was performed with Iview DAB detection kit (Catalog number 760–091, Ventana, Tucson, AZ). The cut off for positive staining is at least 5% of cells with moderate or strong intensity staining for napsin A. Only cytoplasmic dot staining was recognized as positive stain. When evaluating the TMA, the immunopositivity for macrophages and “edge effect”, with positivity seen at the edges of the TMA and negativity at the center of the TMA, should be excluded [9].

## Results

### Expression of napsin a in renal neoplasms

**Figure 1:**
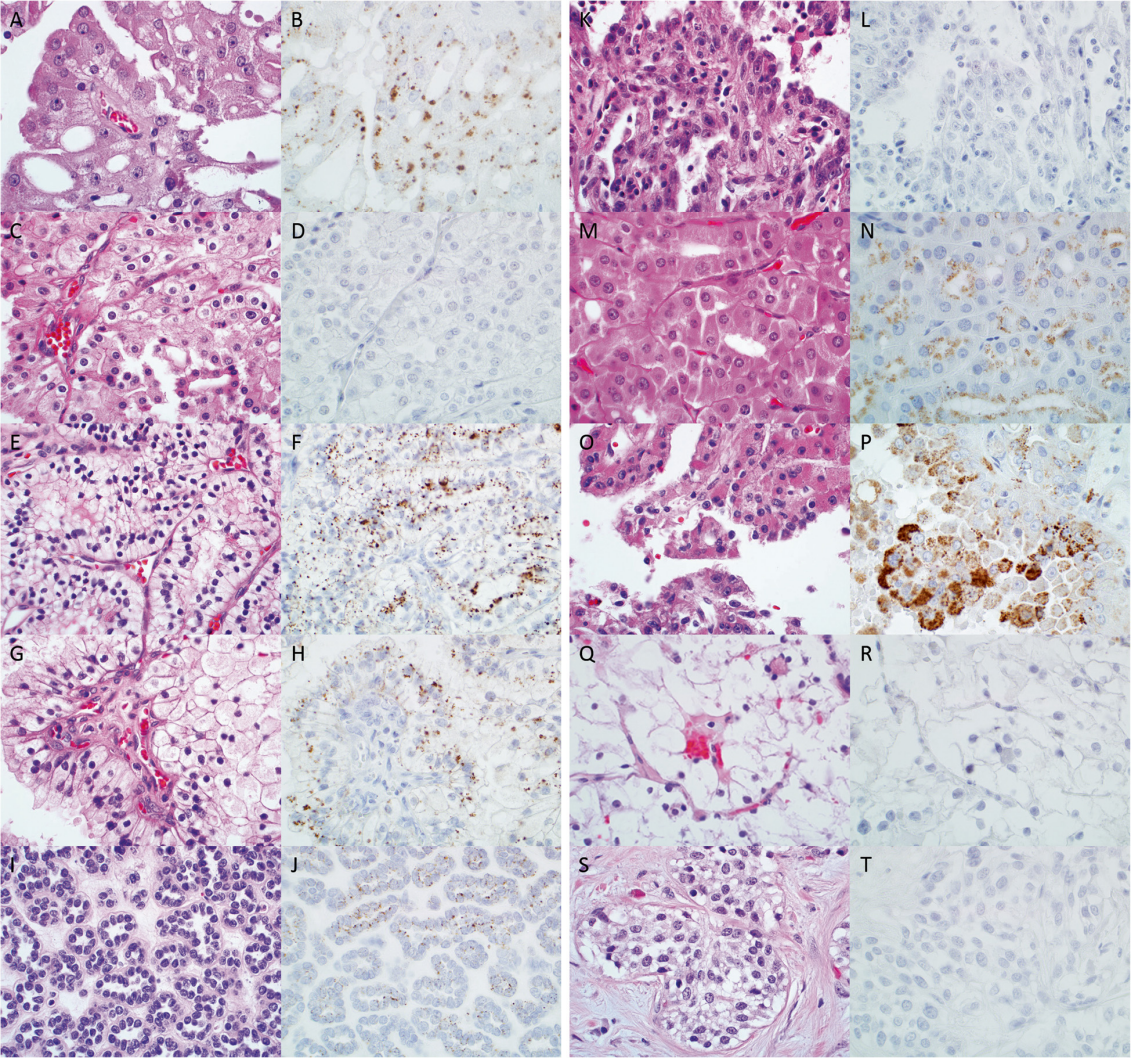
**Expression of napsin A in various renal neoplasms.** Photos of H&E stained slides **(A, C, E, G, I, K, M, O, Q,** and **S)** and **IHC** for napsin **A (B, D, F, H, J, L, N, P, R,** and **T)** of Acquired cystic disease associated RCC **(A** and **B)**, Chromophobe RCC **(C** and **D)**, Clear cell RCC **(E** and **F)**, Clear cell papillary RCC **(G** and **H)**, Metanephric adenoma **(I** and **J)**, Mucinous tubular and spindle cell carcinoma **(K** and **L)**, Oncocytoma **(M** and **N)**, Papillary RCC **(O** and **P)**, TFE/MITF RCC **(Q** and **R)**, and Urothelial carcinoma **(S** and **T)**.

Expression of napsin A was detected in most of renal neoplasms with variable frequency (Table 1 and Figure 1). High frequency of napsin A expression was found in acquired cystic disease associated RCC (100.0%), metanephric adenoma (100.0%), oncocytoma (56.5%) and papillary RCC (83.8%). For metanephric adenoma, the granular cytoplasmic labeling was subtle relative to other tumor types due to scant nature of the cytoplasm in metanephric adenomas. Low frequency of napsin A expression was found in chromophobe RCC (11.1%), clear cell RCC (43.5%), and clear cell papillary RCC (47.4%). Expression of napsin A was not detected in mucinous tubular and spindle cell carcinoma, TFE/MITF RCC, or urothelial carcinoma of the renal pelvis.

## Discussion

In this study, we found that napsin A is expressed in various types of renal neoplasms with variable sensitivities. Our results may have implications in various clinical settings, including the evaluation of needle core biopsies of renal masses and the work up of metastatic carcinomas from unknown primary sites.

We found that immunoreactivity for napsin A was seen in all acquired cystic disease associated RCC and metanephric adenomas (100%). Approximately one-half (47.4%) of the recently described entity clear cell papillary RCC also labeled for Napsin A. Immunoreactivity for napsin A was not seen in mucinous tubular and spindle cell carcinoma or TFE/MITF RCC. To the best of our knowledge, no study has previously reported on the immunoreactivity of Napsin A in these specific renal tumor types. Therefore, IHC for napsin A may help to distinguish these renal neoplasms, especially on renal biopsy specimens.

In this study, we evaluated the immunoreactivity of napsin A in a larger number of oncocytomas and chromophobe RCC and found that immunoreactivity for napsin A was seen in 56.5% oncocytoma, which is different from a previous report (0%) [3]. This is probably due to the inclusion of larger number of cases in our study (23 vs. 3 cases), and the different antibodies used in the studies (Biocare Medical vs. Novocastra) that may result in difference in staining pattern due to different affinity to the antigen. Immunoreactivity for napsin A was detected in 11.1% chromophobe RCC in our study, which falls within the range of previously reported napsin A immunoreactivity (2.9 – 20%) in this tumor type [3,4]. The huch higher rate of 20% immunoreactivity for napsin A in chromophobe RCC previously reported may be due to the limited number of cases used in the prior study (5 cases vs. 45 cases in our study) [3]. The finding of relative overexpression of napsin A in oncocytomas versus chromophobe RCC (56.5% versus 11%) may be helpful in distinguishing these two tumor types from one another.

In this study, we found that immunoreactivity for napsin A was present in several renal neoplasms with similar frequencies as those reported previously. Immunoreactivity for napsin A was detected in 43.5% clear cell RCC, which is in the range of previously reported sensitivity (29.4 - 52%) [3,4,7]. We also found that immunoreactivity for napsin A was seen in 83.8% papillary RCC, which is similar to prior published studies (79.0 - 87.5%) [3,4,7]. We did not observe immunoreactivity for napsin A in urothelial carcinomas of the renal pelvis, a finding which has been noted by others [7].

Traditionally, all localized solid renal masses have been considered potentially malignant and treated with surgical excision, most often radical nephrectomy, in an effort to minimize the risk of metastatic dissemination [10]. However, renal biopsy has a definite and expanding role in the evaluation and treatment of renal masses. Clinically, one of the most obvious uses of biopsy of kidney masses is to distinguish a primary renal neoplasm from a metastatic malignancy. Based on our results, if the biopsied tumor is positive for napsin A, the differential diagnosis is broad and should include both primary renal tumors and metastatic tumors. Immunoreactivity for napsin A can be seen in lung adenocarcinoma [3-7], papillary thyroid carcinoma [3,4], hepatocellular carcinoma [6,7], and endometrial adenocarcinoma [3,7] as well as the renal tumor subtypes identified in this study. In general, if immunohistochemistry is required when evaluating a needle core biopsy or cellblocks of a renal mass a broad panel should be employed. A recent article highlighted the utility of a panel that includes CA IX, CD10, AMACR, CK7, and CD117 if the differential diagnosis is limited to common primary renal tumors—such as clear cell RCC, papillary RCC, and chromophobe RCC [11]. If the differential diagnosis of a tumor sampled by needle core biopsy includes a metastatic carcinoma—such as a lung metastasis, then additional markers such as PAX8, TTF-1, and napsin A can be employed with the caveat that none of these markers are 100% specific or sensitive for a given diagnosis. When dealing with a napsin A positive metastatic carcinoma of unknown primary involving the lung, bone, liver, or other sites the immunoprofile must be considered in the context of the clinical and radiographic history. Essentially, the reader should be wary of basing a diagnosis of site of origin solely on napsin A labeling. Additionally it is important to be aware of the fact that RCC frequently metastasizes to the lung and that the majority of pulmonary metastases of RCC are of the clear cell type. In our study, we found that 47.5% of clear cell RCCs were positive for Napsina A. When dealing with a tumor in the lung if the differential diagnosis includes lung adenocarcinoma versus renal cell carcinoma including clear cell RCC, IHC for TTF-1, PAX-8, and vimentin in addition to napsin A may be helpful. Finally, based on prior reports nonpulmonary napsin A-positive tumors generally stain weakly positive when compared to lung adenocarcinoma. In addition, the presence of macrophages and background staining should be considered when interpreting results [7]. One should also be aware of the existence of TTF-1 negative, napsin A-positive pulmonary adenocarcinoma.

We included a few uncommon renal tumors in the study. Napsin A was immunoreactive in 2 of 2 (100%) acquired cystic disease associated RCC, 3 of 3 (100%) metanephric adenoma, 0 of 1 (0%) mucinous tubular and spindle cell carcinoma, and 0/1 (0%) TFE/MITF RCC. The definitive conclusion for Napsina A expression in these rare tumors with small case number needs further studies by inter-institutional collaboration in future.

## Conclusions

In summary, our results show that napsin A is expressed in a broad spectrum of renal neoplasms with varying frequency. When a metastatic carcinoma of unknown primary is positive for napsin A, the differential diagnosis should include tumors of both renal and lung origin. if both renal cell carcinoma and lung adenocarcinoma are in the differential diagnosis based on morphology and/or clinical history a broad IHC panel including TTF-1, PAX-8 and vimentin should be applied for a definitive diagnosis. Napsin A may be a helpful marker in the differential diagnosis of oncocytoma and chromophobe RCC and further studies on this topic with a larger number of cases is warranted.
